# Traditional Chinese medicine active ingredients-based selenium nanoparticles regulate antioxidant selenoproteins for spinal cord injury treatment

**DOI:** 10.1186/s12951-022-01490-x

**Published:** 2022-06-14

**Authors:** Siyuan Rao, Yongpeng Lin, Rui Lin, Jinggong Liu, Hongshen Wang, Weixiong Hu, Bolai Chen, Tianfeng Chen

**Affiliations:** 1 Guangzhou University of Chinese Medicine, Guangzhou, 510006 China; 2 Division of Spine Center, The Second Affiliated Hospital of Guangzhou University of Chinese Medicine, Guangzhou, 510120 China; 3 Department of Chemistry, Jinan University, Guangzhou, 510632 China

**Keywords:** Traditional Chinese Medicine active ingredients, Selenium nanoparticles, Antioxidant selenoproteins, Spinal cord injury

## Abstract

**Background:**

As Traditional Chinese Medicine (TCM) drugs, Huangqi and Danshen are always applied in combination for spinal cord injury (SCI) treatment based on the compatibility theory of TCM. Astragalus Polysaccharidesis (APS) and Tanshinone IIA (TSIIA) are the main active ingredients of Huangqi and Danshen, and they both possess neuroprotective effects through antioxidant activities. However, low solubility and poor bioavailability have greatly limited their application. In recent years, selenium nanoparticles (SeNPs) have drawn enormous attention as potential delivery carrier for antioxidant drugs.

**Results:**

In this study, TCM active ingredients-based SeNPs surface decorated with APS and loaded with TSIIA (TSIIA@SeNPs-APS) were successfully synthesized under the guidance of the compatibility theory of TCM. Such design improved the bioavailability of APS and TSIIA with the benefits of high stability, efficient delivery and highly therapeutic efficacy for SCI treatment illustrated by an improvement of the antioxidant protective effects of APS and TSIIA. The in vivo experiments indicated that TSIIA@SeNPs-APS displayed high efficiency of cellular uptake and long retention time in PC12 cells. Furthermore, TSIIA@SeNPs-APS had a satisfactory protective effect against oxidative stress-induced cytotoxicity in PC12 cells by inhibiting excessive reactive oxygen species (ROS) production, so as to alleviate mitochondrial dysfunction to reduce cell apoptosis and S phase cell cycle arrest, and finally promote cell survival. The in vivo experiments indicated that TSIIA@SeNPs-APS can protect spinal cord neurons of SCI rats by enhancing GSH-Px activity and decreasing MDA content, which was possibly via the metabolism of TSIIA@SeNPs-APS to SeCys_2_ and regulating antioxidant selenoproteins to resist oxidative stress-induced damage.

**Conclusions:**

TSIIA@SeNPs-APS exhibited promising therapeutic effects in the anti-oxidation therapy of SCI, which paved the way for developing the synergistic effect of TCM active ingredients by nanotechnology to improve the efficacy as well as establishing novel treatments for oxidative stress-related diseases associated with Se metabolism and selenoproteins regulation.

**Supplementary Information:**

The online version contains supplementary material available at 10.1186/s12951-022-01490-x.

## Background

Traditional Chinese Medicine (TCM) is a medical science with a unique and complete theoretical system [1]. The syndrome of Qi deficiency and blood stasis is one of the basic TCM syndromes, which is common among different diseases, including spinal cord injury (SCI), cardiovascular and cerebrovascular diseases [2–4]. Accordingly, supplementing Qi and activating blood circulation is used as a treatment principle in the case of QDBS syndrome [5, 6]. Since Huangqi (*Astragali Radix*) and Danshen (*Salvia miltiorrhiza Bunge*) are the representative drugs for supplementing Qi and activating blood circulation respectively, they are always applied in combination based on TCM compatibility theory [7–9]. Such TCM prescription composed of multiple medicinal herbs has shown its synergistic effect through multi-ingredients, multi-targets and multi-pathways [10, 11]. It has been reported that Astragalus Polysaccharidesis (APS) and Tanshinone IIA (TSIIA), the main active ingredients from Huangqi and Danshen respectively, both possess neuroprotective effects through antioxidant activities [12, 13], which have certain effects on neurological disease associated with oxidative stress, such as SCI [14, 15]. However, low solubility and poor bioavailability have greatly limited their application [16, 17]. Hence, it is urgent to explore a novel approach to conquer above problems to maximize the antioxidant effect of APS and TSIIA. Nanotechnology has been considered to be an effective method to improve the effectiveness of the existing drugs [18–22].

In recent years, selenium nanoparticles (SeNPs) have drawn enormous attention in biomedicine thanks to the advantages of low toxicity, good biocompatibility and antioxidation ability, which have been considered as potential delivery carrier for antioxidant or anti-inflammation drugs [23–29]. Our previous study indicated that functionalized SeNPs showed neuroprotective function through antioxidative and anti-inflammatory activities [30]. We also preliminarily demonstrated the therapeutic effect of SeNPs in SCI model [31]. Nevertheless, the potential metabolism and antioxidative mechanism of SeNPs in SCI remains unclear. Selenium (Se) is a component of selenoproteins in the form of selenocysteine (SeCys_2_), playing a critical role in regulating biological processes [32]. There are at least twenty-five human selenoproteins and most of them serves as antioxidant enzymes to alleviate damage caused by reactive oxygen species (ROS) [33]. Previous research has demonstrated that antioxidant selenoproteins are involved in the prevention of central nervous system related diseases [34]. However, whether SeNPs protect SCI from oxidative stress through selenoproteins regulation has not been previously examined.

**Fig. 1:**
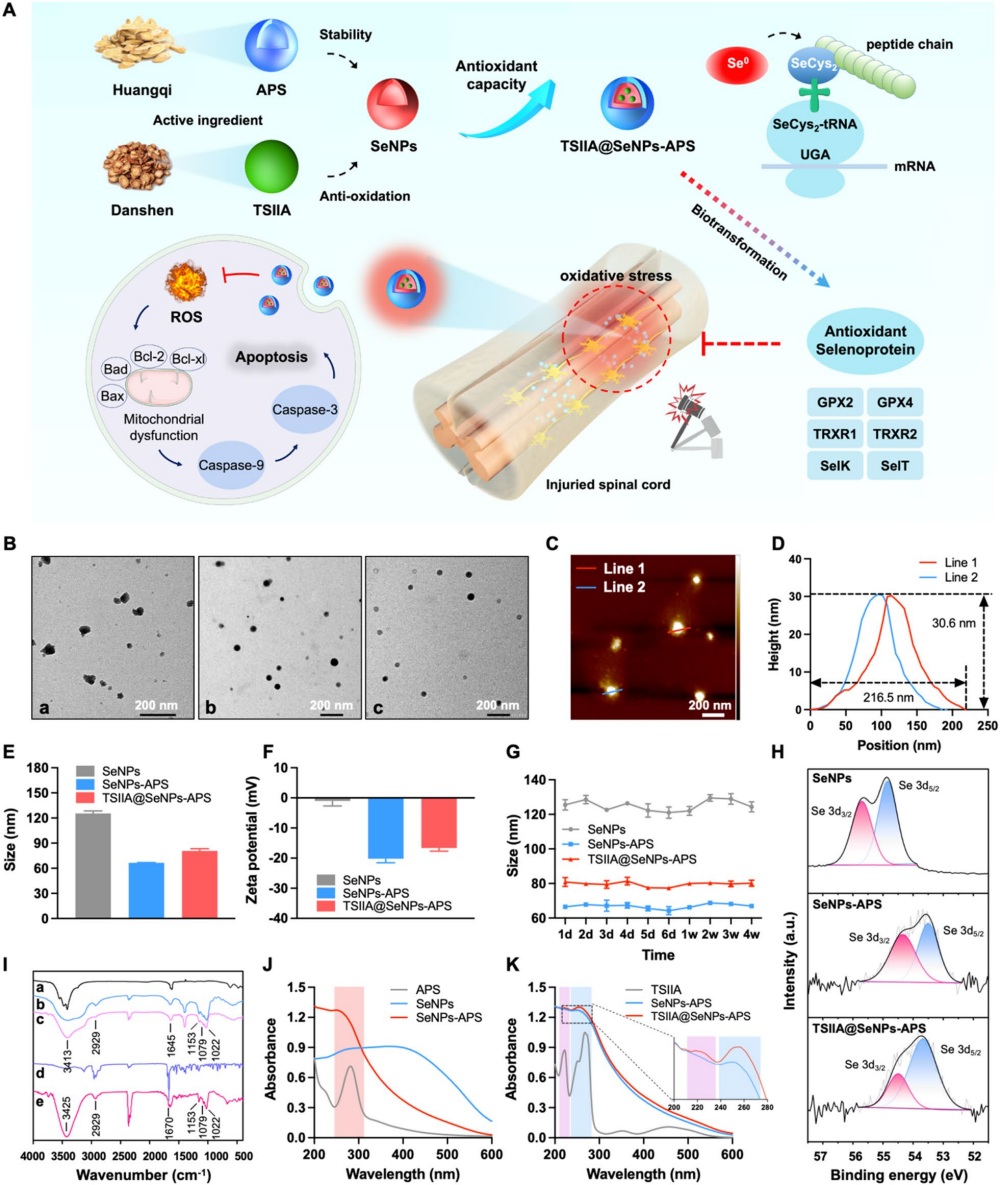
Design of TSIIA@SeNPs-APS based on compatibility theory of TCM. **A** Synthetic schematic diagram of TSIIA@SeNPs-APS and its regulation of antioxidant selenoproteins for SCI treatment. **B** TEM images of (a) SeNPs, (b) SeNPs-APS and (c) TSIIA@SeNPs-APS. The scale bar is 200 nm. **C** AFM image of TSIIA@SeNPs-APS. **D** Corresponding thickness profile of TSIIA@SeNPs-APS in **C**. **E** Size distribution and **F** Surface charge of SeNPs, SeNPs-APS and TSIIA@SeNPs-APS in aqueous solutions. **G** The particle size distribution changes of SeNPs, SeNPs-APS and TSIIA@SeNPs-APS as time progresses. **H** Se 3d XPS pattern of SeNPs, SeNPs-APS and TSIIA@SeNPs-APS. **I** FT-IR spectra of (a) SeNPs, (b) APS, (c) SeNPs-APS, (d) TSIIA and (e) TSIIA@SeNPs-APS. **J–K** UV–Vis spectra of SeNPs, APS, SeNPs-APS, TSIIA and TSIIA@SeNPs-APS

In this study, TCM active ingredients-based SeNPs surface decorated with APS and loaded with TSIIA (TSIIA@SeNPs-APS) were designed inspired by compatibility theory of TCM to synergistically enhance the antioxidant activity of the drugs (Fig. 1A). Additionally, we explored the potential regulation of TSIIA@SeNPs-APS on antioxidant selenoproteins in SCI treatment.

## Results and discussion

### Design of TSIIA@SeNPs-APS based on compatibility theory of TCM

In this study, three kinds of selenium nanoparticles including SeNPs, SeNPs-APS, and TSIIA@SeNPs-APS were fabricated successfully. SeNPs without surface stabilizer were unstable and easy to aggregate. According to the transmission electron microscopy (TEM) images (Fig. 1B), the morphologies of SeNPs were irregular and inhomogeneous. While compared with SeNPs, SeNPs-APS and TSIIA@SeNPs-APS both showed good monodispersity and spherical after the APS surface modification. It could also be seen that the morphologies of SeNPs-APS and TSIIA@SeNPs-APS were similar, which showed that loading TSIIA did not affect the change of the morphologies of SeNPs-APS. Atomic force microscopy (AFM) further investigated the spherical structure of TSIIA@SeNPs-APS in homogeneous distribution (Fig. 1C), with a thickness of about 30.6 nm (Fig. 1D). In addition, we analysed the particle sizes (Fig. 1E) and Zeta potential (Fig. 1F) of the selenium nanoparticles. The particle sizes of SeNPs-APS and TSIIA@SeNPs-APS were 66.5 ± 0.3 nm and 80.8 ± 2.5 nm respectively, which were much smaller than that of SeNPs (125.5 ± 3.0 nm). Besides, the size of TSIIA@SeNPs-APS was slightly larger than that of SeNPs-APS, probably due to the effect of TSIIA loading. The Zeta potential of SeNPs was − 1.0 ± 1.7 mV, close to neutral. However, when SeNPs-APS and TSIIA@SeNPs-APS was formed, their Zeta potentials were − 20.2 ± 1.3 and − 16.6 ± 1.1 mV, respectively, revealing an absolute value increasement, owing to the strong negative electricity of APS. Furthermore, the stability of the selenium nanoparticles in aqueous solution was evaluated by examining the size change (Fig. 1G). It was found that the size change of SeNPs fluctuated between 121 to 130 nm within 4 weeks. However, the particle size of SeNPs-APS and TSIIA@SeNPs-APS remained around 66 and 80 nm, respectively, revealing good stability. These results implied that APS improved the stability of TSIIA@SeNPs-APS in aqueous solution, which have the potential for use in biomedical applications.

To verify the chemical structure of TSIIA@SeNPs-APS, X-ray photoelectron spectroscopy (XPS), fourier transform infrared (FT-IR) spectroscopy and ultraviolet–visible (UV–Vis) absorption spectrum were performed. As shown in Fig. 1H, the peaks of Se 3d_3/2_ and 3d_5/2_ in the Se 3d spectrum shifted from 55.7 and 54.8 eV (SeNPs) to 54.3 and 53.5 eV (SeNPs-APS) and 54.5 and 53.7 eV (TSIIA@SeNPs-APS), respectively, indicating that there was an interaction between APS and Se to make the SeNPs more stable, and the valence state of Se in TSIIA@SeNPs-APS was in elementary status. The FT-IR spectra of TSIIA@SeNPs-APS revealed a peak at 3425 cm^−1^ attributed to the -OH stretching vibration, a peak at 2929 cm^−1^ attributed to the C-H stretching vibration, and three characteristic bands at 1153, 1079 and 1022 cm^−1^ attributed to the asymmetric vibrations of C–O–C glycosidic rings, which were characteristic peak of APS [35]. Besides, the main characteristic peak of TSIIA could be found at 1670 cm^−1^ designated to the C = O stretching vibration in the spectrum of TSIIA@SeNPs-APS (Fig. 1I) [36]. The UV–Vis spectrum also showed that the absorption peak of APS at 281 nm and the absorption peaks of TSIIA at 224 nm and 268 nm were found in the spectra of TSIIA@SeNPs-APS (Fig. 1J–K). These results implicated TSIIA@SeNPs-APS were fabricated successfully with modification by APS and loading TSIIA.

### Cellular uptake and intracellular trafficking of TSIIA@SeNPs-APS

**Fig. 2:**
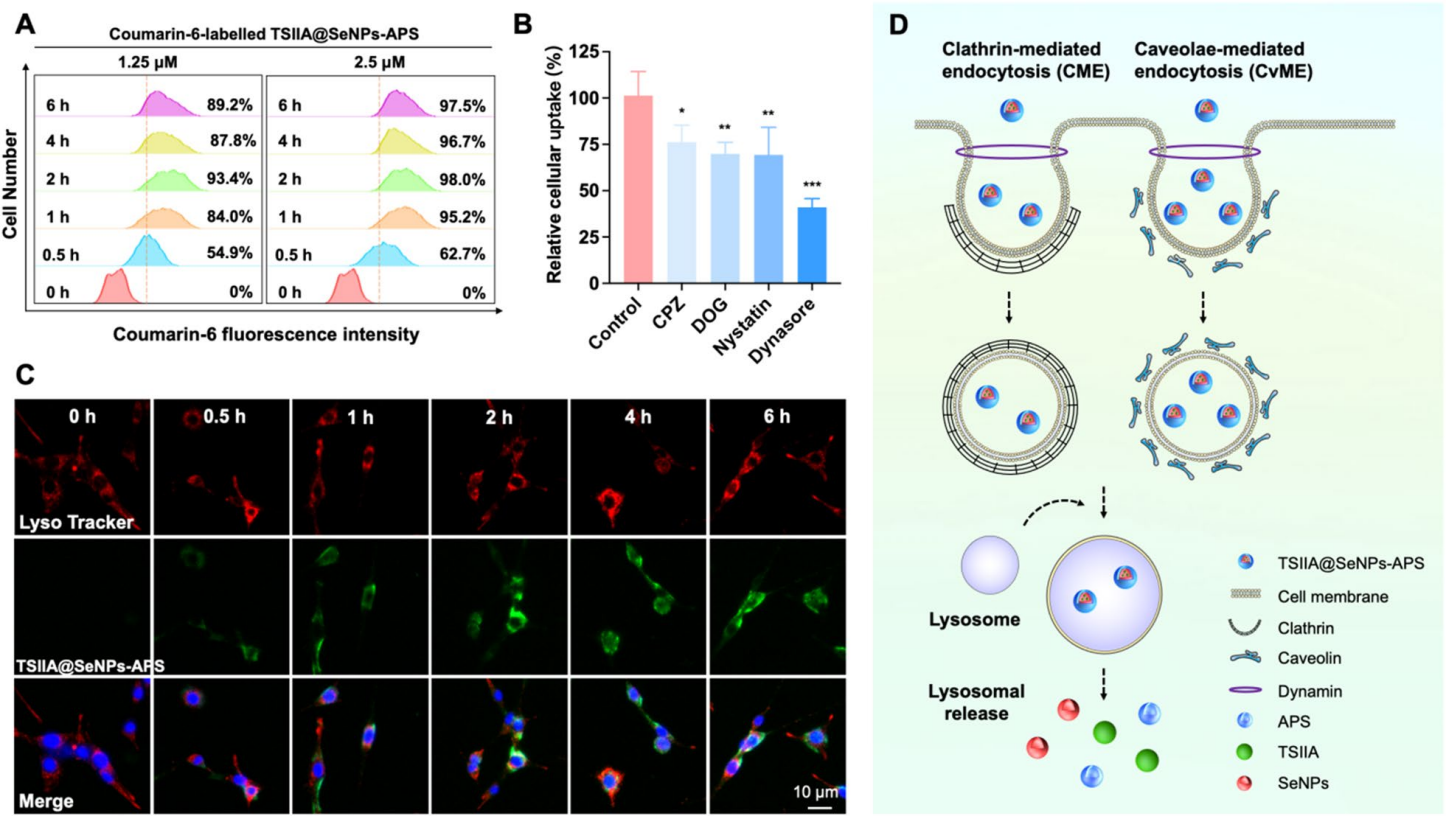
Cellular uptake and intracellular trafficking of TSIIA@SeNPs-APS. **A** Fluorescence intensity of couramin-6-labelled TSIIA@SeNPs-APS in PC12 cells for different time assessed by flow cytometry. **B** Cellular uptake of couramin-6-labelled TSIIA@SeNPs-APS in PC12 cells after pretreated with different inhibitors. Significant difference between control group and other groups is indicated by *P < *0.05*, **P < *0.01*, ***P < *0.001*. **C** Intracellular trafficking of couramin-6-labelled TSIIA@SeNPs-APS in PC12 cells, which were stained by DAPI (blue, nucleus) and Lysotracker (red, lysosome), and visualized using a fluorescence microscopy. The scale bar is 10 nm. **D** Schematic illustration of cellular uptake and intracellular trafficking of TSIIA@SeNPs-APS

High efficiency of cellular uptake and long retention time in cells is crucial for nanoparticles as delivery carriers [37]. Thus, we synthesized the couramin-6-labelled TSIIA@SeNPs-APS to explore its cellular uptake and intracellular trafficking. Fluorescence intensity of couramin-6-labelled TSIIA@SeNPs-APS in PC12 cells was assessed at multiple time points for cellular uptake detection (Fig. 2A). The results showed the cellular uptake of TSIIA@SeNPs-APS increased as the time and the concentration increased. with the highest accumulation in 2 h and maintained the high cellular uptake level within 6 h.

There are two major endocytic pathways for internalization of nanocarriers: clathrin-mediated endocytosis (CME) and caveolae-mediated endocytosis (CvME) [38]. Hence, in order to investigate the mechanisms of endocytosis of TSIIA@SeNPs-APS, PC12 cells were preincubated with different inhibitors, including chlorpromazine (CPZ) and 2-deoxy-D-glucose (DOG) for CME inhibition, nystatin for CvME inhibition and dynasore for the inhibition of both CME and CvME. As shown in Fig. 2B, these endocytosis inhibitors evidently inhibited the internalization of TSIIA@SeNPs-APS as the cellular uptake efficiency decreased to 76.2%, 69.8% 69.3% and 41.0% after CPZ, DOG, nystatin and dynasore treatments, respectively. These results demonstrated that both CME and CvME were the major pathways of the internalization of TSIIA@SeNPs-APS by PC12 cells.

To visualize the trafficking of TSIIA@SeNPs-APS within PC12 cells, TSIIA@SeNPs-APS were labelled by coumarin-6 (green) as a fluorescent marker and PC12 cells were stained with LysoTracker and DAPI (Fig. 2C). It can be seen from the overlapping part of fluorescence that TSIIA@SeNPs-APS was first located in lysosomes within 0.5 h. Then TSIIA@SeNPs-APS accumulated continuously in lysosomes that the fluorescence intensity became stronger and reached the maximum in 2 h. These results were consistent with the results of cellular uptake. After 4 h incubation, TSIIA@SeNPs-APS dispersed in the whole cytoplasm. These results suggested that after internalized by endocytosis, TSIIA@SeNPs-APS were transported to lysosomes and released from lysosomes (Fig. 2D).

### Protection of PC12 cells by TSIIA@SeNPs-APS against oxidative stress damage

**Fig. 3:**
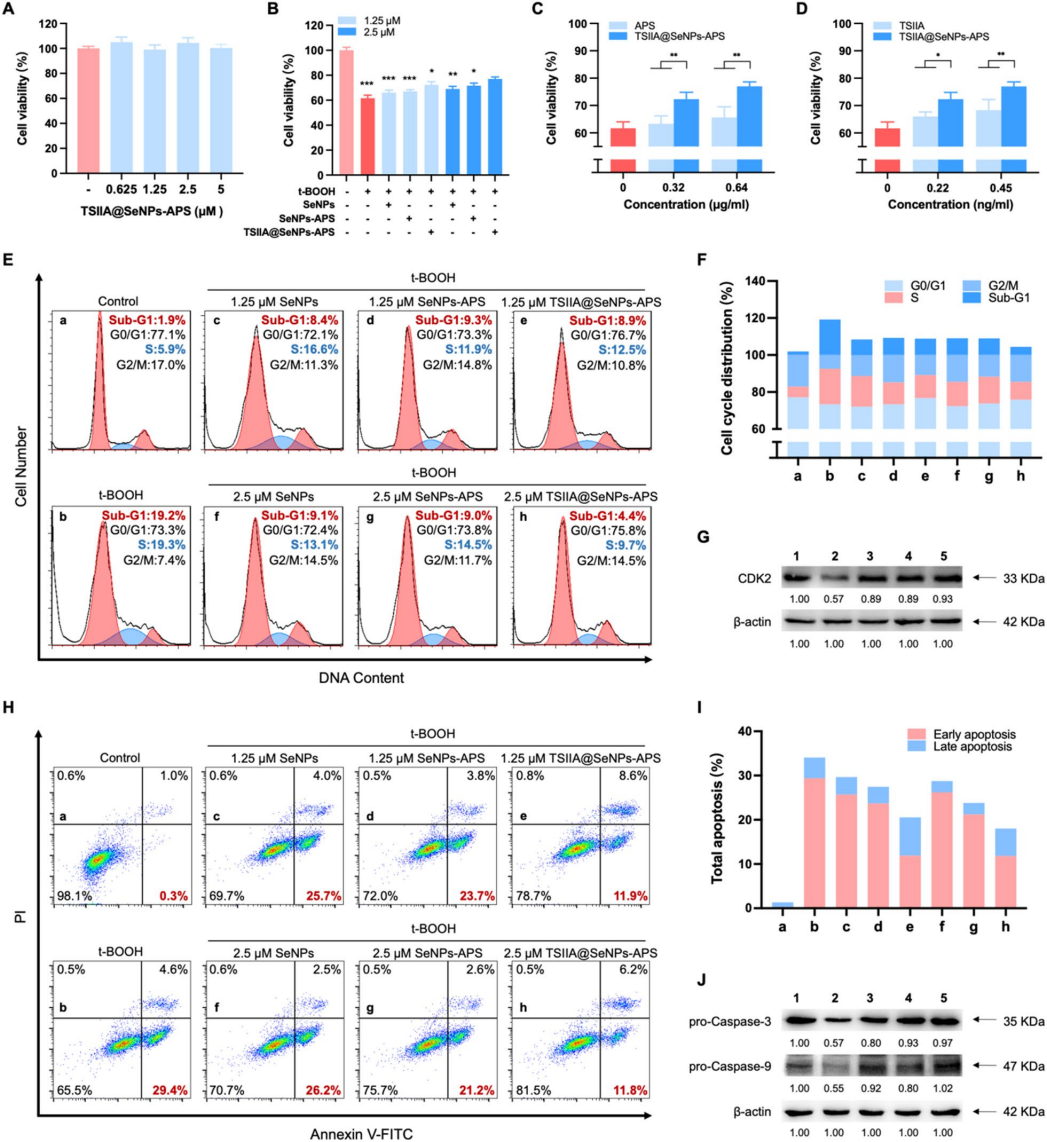
Effects of t-BOOH on cell viability, apoptosis and cell cycle of PC12 cells reversed by TSIIA@SeNPs-APS. **A** The viability of PC12 cells under different concentrations of TSIIA@SeNPs-APS for 24 h (calculated by Se concentration). **B** The viability of PC12 cells pretreated with t-BOOH and then treated with SeNPs, SeNPs-APS and TSIIA@SeNPs-APS for 24 h, respectively, at the concentration of 1.25 and 2.5 μM (calculated by Se concentration). Significant difference between the 2.5 μM TSIIA@SeNPs-APS group and the other groups is indicated by *P < *0.05*, **P < *0.01*, ***P < *0.001*. **C** The viability of PC12 cells pretreated with t-BOOH and then treated with APS and TSIIA@SeNPs-APS for 24 h, respectively, at the concentration of 0.32 and 0.64 μg/ml (calculated by APS concentration). **P < *0.01*. **D** The viability of PC12 cells pretreated with 100 μM t-BOOH and then treated with TSIIA and TSIIA@SeNPs-APS for 24 h, respectively, at the concentration of 0.22 and 0.45 ng/ml (calculated by TSIIA concentration). *P < 0.05, **P < *0.01*. **E** Flow cytometry analysis of the effects of SeNPs, SeNPs-APS and TSIIA@SeNPs-APS on the cell cycle distribution of PC12 cells pretreated with t-BOOH. **F** Proportion of the cell cycle in (**E**). **G** Western blot analysis of CDK2. Lane 1–5: groups of control, t-BOOH, t-BOOH + SeNPs, t-BOOH + SeNPs-APS and t-BOOH + TSIIA@SeNPs-APS, respectively. **H** Annexin V-FITC/PI double-staining to evaluate the effects of SeNPs, SeNPs-APS and TSIIA@SeNPs-APS on the apoptosis of PC12 cells pretreated with t-BOOH. **I** Quantitative analysis of the proportion of apoptosis in (**H**). **J** Western blot analysis of pro-Caspase-3 and pro-Caspase-9. Lane 1–5: the same as groups in **G**

The PC12 cell line has been widely applied as a model to explore neuronal injury diseases and neurodegenerative diseases [39]. Thus, tert-butyl hydroperoxide (t-BOOH), an oxidizing agent, was used as an oxidative stress damage induction to PC12 cells in order to develop an in vitro model of SCI and verify the protective effects of TSIIA@SeNPs-APS in PC12 cells. Nano selenium is attracting much attention due to its lower toxicity than inorganic and organic selenium, so we first assessed the safe dosage range of TSIIA@SeNPs-APS. The results revealed that there was no cytotoxicity of TSIIA@SeNPs-APS on PC12 cells within the concentration range of 0–5 μM (Fig. 3A). As shown in Fig. 3B , 100  μM t-BOOH reduced the viability of PC12 cells to 61.7%, which could be reversed by SeNPs, SeNPs-APS and TSIIA@SeNPs-APS, respectively. Especially the treatment of TSIIA@SeNPs-APS at the Se concentration of 2.5 μM evidently raised the viability of PC12 cells from 61.7% to 77.0%. The effects of APS and TSIIA on PC12 cells were also studied for complete comparison. As calculated by APS or TSIIA concentration, the viability of TSIIA@SeNPs-APS was significantly higher than that of APS or TSIIA (Fig. 3C, D). Taken together, we concluded that the protective effects of APS and TSIIA in PC12 cells have been improved after nano-preparation. In addition, APS modification and TSIIA loading also enhanced the ability of SeNPs to preserve the viability of PC12 cells against oxidative stress damage.

To study the possible protective mechanism of TSIIA@SeNPs-APS in PC12 cells, we assessed the cell apoptosis and cell cycle arrest, which are widely believed to be the major mechanism of cell death induction and cell growth inhibition triggered by oxidative stress [40]. As shown in Fig. 3E, F, pretreatment with t-BOOH significantly increased the apoptosis and S phase cell cycle arrest of PC12 cells, as evidenced by the increased proportion of Sub-G1 from 1.9 to 19.2% and S phase from 5.9% to 19.3%. It can be seen that TSIIA@SeNPs-APS at the concentration of 2.5 μM significantly counteracted the t-BOOH-induced increase in Sub-G1 population from 19.2 to 4.4% and S phase population from 19.3 to 9.7%. These results were further confirmed by western blot analysis (Fig. 3G), showing that TSIIA@SeNPs-APS treatment increased the expression of S phase related proteins CDK2, which were down regulated by t-BOOH pretreatment. Flow cytometric analysis of Annexin V-FITC/PI staining was then conducted for further verifying the change of apoptosis process. As shown in Fig. 3H, I, t-BOOH markedly increased the percentage of early apoptotic cells from 0.3% to 29.4%, which could be declined remarkably from 29.4% to 11.8% by TSIIA@SeNPs-APS at the concentration of 2.5 μM. Furthermore, western blot analysis manifested that TSIIA@SeNPs-APS decreased the cleavage of apoptotic related proteins Caspase-3 and Caspase-9 in t-BOOH-treated PC12 cells (Fig. 3J), which further demonstrated the anti-apoptosis effect of TSIIA@SeNPs-APS was mediated via inhibition of mitochondria-mediated apoptotic pathways. Collectively, these results indicated that TSIIA@SeNPs-APS protected PC12 cells from apoptosis and S phase cell cycle arrest caused by oxidative stress damage.

**Fig. 4:**
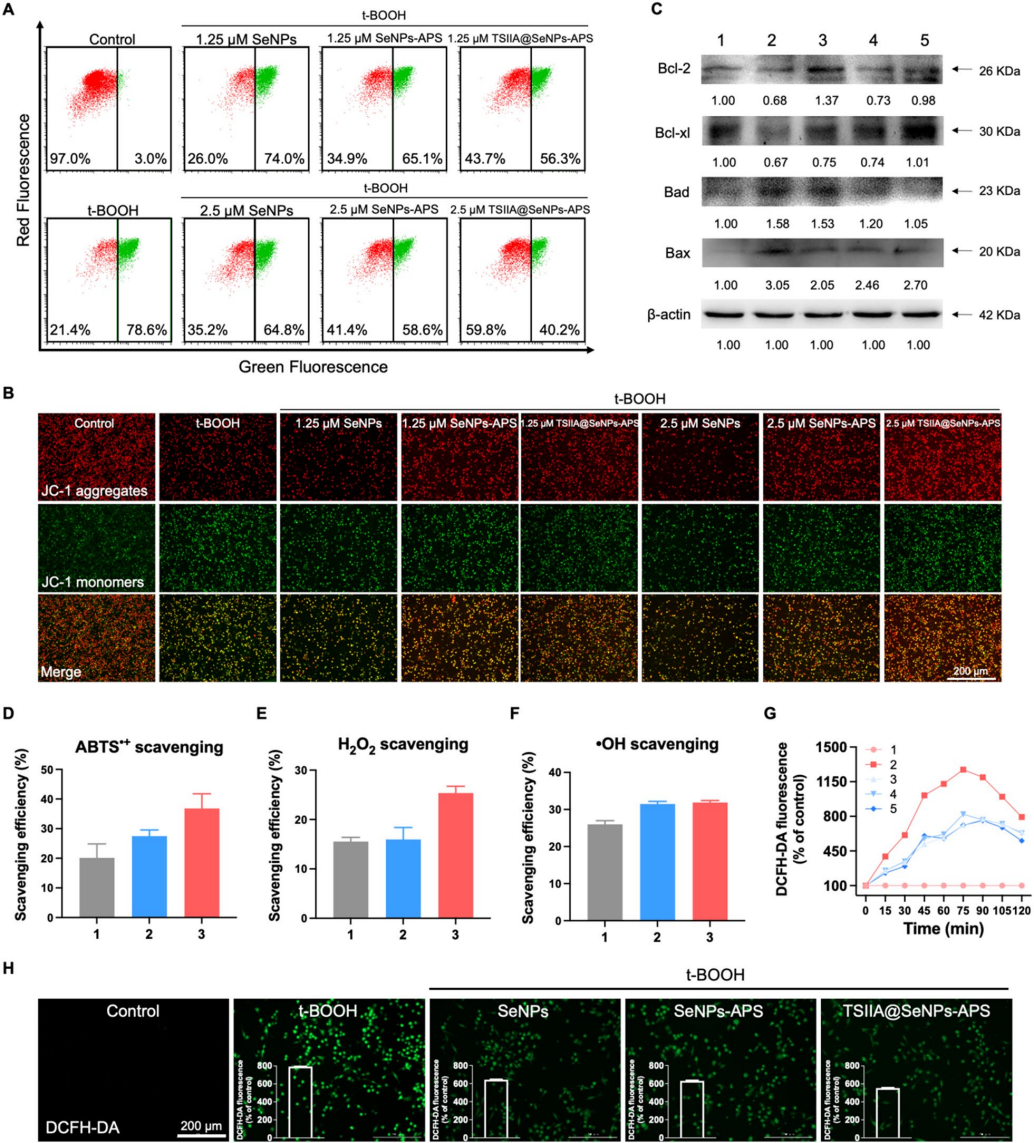
Protective effects of TSIIA@SeNPs-APS against oxidative stress-induced mitochondrial dysfunction in PC12 cells. **A** The ΔΨm of PC12 cells, which were pretreated with 100 μM t-BOOH for 2 h and then treated with SeNPs, SeNPs-APS and TSIIA@SeNPs-APS for 24 h, examined by JC-1 staining and flow cytometry. **B** JC-1 fluorescence images of PC12 cells in **A**. **C** Western blot analysis of Bcl-2 family proteins. Lane 1–5: groups of control, t-BOOH, t-BOOH + SeNPs, t-BOOH + SeNPs-APS and t-BOOH + TSIIA@SeNPs-APS, respectively. **D–F** ROS scavenging efficiency of (1) SeNPs, (2) SeNPs-APS and (3) TSIIA@SeNPs-APS for ABTS^•+^, H_2_O_2_ and •OH, respectively. **G** Changes in intracellular total ROS level in PC12 cells, which were examined by DCFH-DA staining in group 1–5: groups of control, t-BOOH, t-BOOH + SeNPs, t-BOOH + SeNPs-APS and t-BOOH + TSIIA@SeNPs-APS, respectively. **H** The fluorescence intensity and representative images of DCFH-DA in PC12 cells at 120 min after treatment

Mitochondrial membrane potential (ΔΨm) is a significant early determinant of the mitochondria-mediated apoptotic pathways as well as an important factor in assessing the function of the mitochondria [41]. Therefore, we examined the ΔΨm of PC12 cells by flow cytometry with the JC-1 dye, which emits red fluorescence in healthy mitochondria (JC-1 aggregates), while emits green fluorescence in depolarized mitochondria (JC-1 monomers). The decreased ratio of red/green fluorescence of JC-1 is an indicator for early stage of cell apoptosis [42]. As shown in Fig. 4A, the proportion of cells with depolarized mitochondria in 1.25 and 2.5 μM TSIIA@SeNPs-APS decreased from 78.6% to 56.3% and 40.2%, respectively. This result was further verified by fluorescence images that the decrease in ratio of red/green fluorescence after t-BOOH pretreatment was restored by TSIIA@SeNPs-APS (Fig. 4B). In addition, western blot analysis revealed that TSIIA@SeNPs-APS significantly promoted the anti-apoptotic proteins expression of Bcl-2 and Bcl-xl and suppressed the pro-apoptotic proteins expression of Bax and Bad, indicating that the mitochondrial dysfunction and apoptosis caused by t-BOOH could be reversed by TSIIA@SeNPs-APS (Fig. 4C).

ROS/oxidative stress plays a significant role in the SCI. Excess ROS such as hydrogen peroxide (H_2_O_2_), hydroxyl radical (•OH) and superoxide (O2^•−^) increases significantly in the spinal cord within hours after the primary injury, which breaks the pro-oxidant/anti-oxidant dynamic balance [43], and causes progressive oxidative stress damage to mitochondria, proteins and DNA resulting in apoptosis and necrosis of neuronal cells, acting as a mechanism of secondary spinal injury [44, 45]. Although the primary injury is irreversible, the secondary injury can be prevented and alleviated by intervention. As consequence, reducing secondary injury through antioxidation has been considered as a promising treatment strategy for SCI [46]. Here, the scavenging efficiency of TSIIA@SeNPs-APS to consume ROS was evaluated by several widely used assays, including ABTS^•+^, H_2_O_2_ and •OH scavenging assays. The total antioxidant activity of TSIIA@SeNPs-APS was evaluated by ABTS method. As shown in Fig. 4D, the ABTS^•+^ scavenging efficiency of TSIIA@SeNPs-APS was about 37.8% at 300 μM, higher than that of SeNPs (20.2%) and SeNPs-APS (27.5%). The total antioxidant activity of APS and TSIIA was improved as evidenced by the increased ABTS^•+^ scavenging efficiency of TSIIA@SeNPs-APS shown in Additional file 1: Fig. S1. Furthermore, Fig. 4E showed that the TSIIA@SeNPs-APS also exhibited a high H_2_O_2_ scavenging efficiency (25.4%) compared with SeNPs (15.5%) and SeNPs-APS (16.0%). In addition, more than 30% of the •OH was scavenged by TSIIA@SeNPs-APS (Fig. 4F). Hence, we concluded that through APS modification and TSIIA loading, the ROS scavenging capability of SeNPs was improved remarkably, particularly in the scavenging of ABTS^•+^ and H_2_O_2_. Furthermore, the ROS scavenging ability of TSIIA@SeNPs-APS in PC12 cells was conducted by 2′,7′-dichlorodihydrofluorescein diacetate (DCFH-DA) staining, a ROS-sensitive fluorescent probe, which could be deacetylated by cellular esterases to the non-fluorescent DCFH, and later oxidized by intracellular ROS to the green fluorescent DCF [47]. As shown in Fig. 4G, ROS in PC12 cells rose to 1271.4% after 75 min of incubation with t-BOOH and maintained a high level around 792.8% at 120 min, as the control group was 100%. Interestingly, the increase of intracellular ROS was significantly suppressed by TSIIA@SeNPs-APS, which declined to 553.6% at 120 min. The representative fluorescence images of PC12 cells showed corresponding results that there was a strong green fluorescence in t-BOOH group while it was weakened after TSIIA@SeNPs-APS treatment (Fig. 4H).

### Improvement of locomotor function and neurons survival by TSIIA@SeNPs-APS in SCI rats

**Fig. 5:**
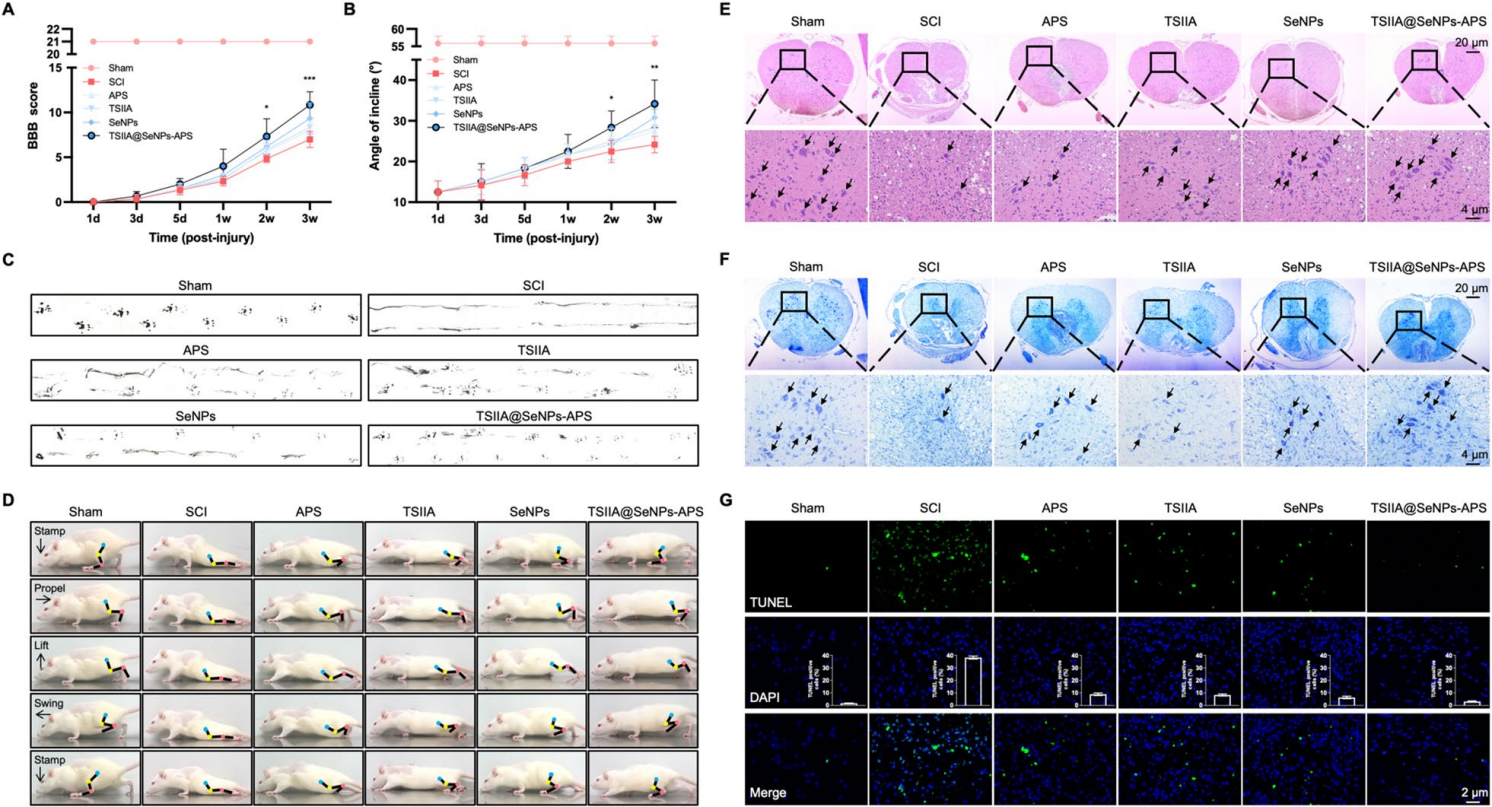
Improvement of locomotor recovery and neurons survival by TSIIA@SeNPs-APS in SCI rats. **A** BBB scores and **B** Inclined plane test of rats at different times. Significant difference between TSIIA@SeNPs-APS group and SCI group is indicated by *P < *0.05*, **P < *0.01* and ***P < *0.001*. **C** Footprint analysis of rats in each group. **D** Snapshots extracted from video recordings showing a sequence of hindlimb movements rats in each group while walking. Iliac crest, knee, and ankle joints were showed by dots and lines. The moving direction of hindlimb was showed by arrow. Representative images of **E** H&E staining, **F** Nissl staining, and **G** TUNEL/DAPI double-staining of spinal cord tissues in each group. Black arrows: motor neurons in **E** and Nissl bodies **in F**

To further evaluate the neuroprotective effectiveness of TSIIA@SeNPs-APS in vivo, we first assessed the recovery of locomotor function of the rats. The Basso-Beattie-Bresnahan (BBB) scores and inclined plane test in each group were evaluated at 1, 3, 5, 7, 14 and 21 days after sham surgery or SCI operation. As shown in Fig. 5A, all rats except those in sham group presented the behavior of paralysis at 1 d, with BBB score of 0. A gradual recovery of BBB scores could be found in all the treatment groups, but the scores of TSIIA@SeNPs-APS group were significantly higher at 14 and 21 days after surgery. TSIIA@SeNPs-APS also improved the angle of incline compared with that in the sham group (Fig. 5B). In footprint analysis, rats in the SCI group showed incongruous gaits and extensive drag of hindlimbs. As expected, the hindlimbs in the TSIIA@SeNPs-APS group showed a marked recovery of gait with an improvement in coordinated motor functions, compared with those in other treatment groups (Fig. 5C). In order to further analyze the function of hindlimbs in rats, we studied the hindlimbs motion during walking, which can be broken down into a sequence of events including the motion of stamp-propel-lift-swing-stamp as a cycle (Fig. 5D). In the SCI group, hindlimbs of the rats were paralyzed and dragged all the time while the improvements could be observed in the TSIIA@SeNPs-APS group, including re-establishment of a standing pose (ability to stamp), better weight support (increased iliac crest height) and recovery of movement in the range of joints motion (ability to propel and swing).

Hematoxylin and eosin (H&E) staining, Nissl staining and TUNEL/DAPI double-staining were applied to detect the effects of TSIIA@SeNPs-APS on histological pathology of spinal cord tissue. The H&E staining revealed the intact structure and normal morphology of the spinal cord in the sham group, with evenly arranged neuronal cells and clear nucleoli. Compared with the sham group, gray structure disorder, obvious necrosis and cavity, irregular neurons and pyknotic nuclei, and a large number of motor neurons lost happened in the SCI group, whereas treatment of TSIIA@SeNPs-APS showed protective effects as evident by above pathological symptoms were improved significantly (Fig. 5E). It was also supported by Nissl staining, which was used to verify the state of neurons [48]. The quantity of the Nissl bodies was reduced markedly in the SCI group, while they were restored and clearly visible in TSIIA@SeNPs-APS treated group (Fig. 5F). Moreover, TUNEL/DAPI double-staining was conducted to determine whether TSIIA@SeNPs-APS could attenuate apoptosis in vivo. As shown in Fig. 5G, there were many TUNEL-positive cells in the SCI group, which was decreased significantly in the TSIIA@SeNPs-APS group. These findings indicated that TSIIA@SeNPs-APS treatment significantly reduced neuronal damage and apoptosis in SCI rats.

### Biotransformation and antioxidant selenoproteins regulation of TSIIA@SeNPs-APS

**Fig. 6:**
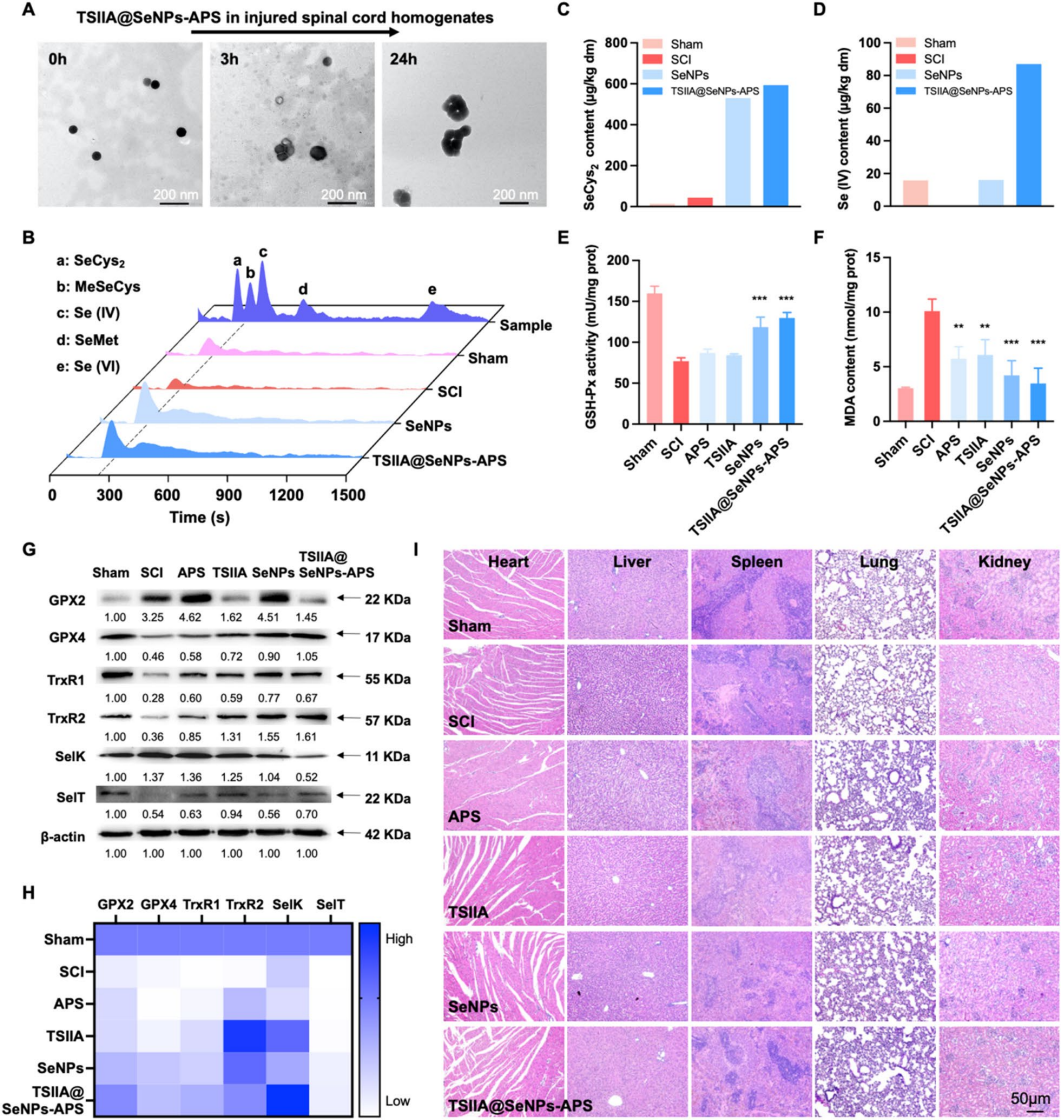
The metabolism and bioactivity mechanism of TSIIA@SeNPs-APS in vivo. **A** TEM images revealing the morphological and structural changes of TSIIA@SeNPs-APS incubated in injured spinal cord homogenates within 24 h. **B** Chromatograms of Se speciation in the spinal cord tissue by HPLC-ICP-MS. Corresponding quantitative analysis of **C** SeCys_2_ and (**D**) SeIV in (**B**). The changes of **E** GSH-Px activity and **F** MDA level in the spinal cord tissue of each group. Significant difference between the SCI group and the other groups is indicated by **P < *0.01* and ***P < *0.001*. **G** Expression of GPX2, GPX4, TRXR1, TRXR2, SelK and SelT protein in the spinal cord tissue detected by Western blot. **H** Relative gene expression of GPX2, GPX4, TRXR1, TRXR2, SelK and SelT detected by qPCR. **I** H&E staining of major organs in different groups for toxicity study in vivo

It was reported that the microenvironment featured by oxidative and acid-enriched was formed in the injured spinal cord [49]. In the present study, TEM was applied to monitor the morphological and structural changes of TSIIA@SeNPs-APS incubated in injured spinal cord homogenates to investigate whether selenium nanoparticles could rapidly respond to the injured spinal cord microenvironment, which hasn't been reported before. According to the results in Fig. 6A, the nanoparticles began to agglomerate together and became larger in 3 h. Then, degradation of TSIIA@SeNPs-APS could be observed and the original spherical shape was no longer maintained in 24 h, which may contribute to the release of the loaded TSIIA and superficial APS at the injured spinal cord site.

To further understand the metabolism and bioactivity mechanism of TSIIA@SeNPs-APS, the Se biotransformation in vivo was detected. In our previous study, we had demonstrated that selenocysteine (SeCys_2_), Se-methylselenocysteine (MeSeCys), selenite [Se (IV)], selenomethionine (SeMet) and selenate [Se (VI)] were the main metabolic products of selenium nanoparticles [30, 50, 51], so these five Se-related metabolic products were quantitatively measured in this study using high performance liquid chromatography-inductively coupled plasma mass spectrometrt (HPLC-ICP-MS). As shown in Fig. 6B, only SeCys_2_ and Se (IV) could be measured in the spinal cord. Additionally, the content of SeCys_2_ was at a high level in the SeNPs and TSIIA@SeNPs-APS groups, compared with the groups without Se treatment (Fig. 6C). Moreover, a high-level content of Se (IV) was detected in the TSIIA@SeNPs-APS group while it was also present in the sham group and the SeNPs group with a relatively lower content (Fig. 6D). Taken together, it suggested that TSIIA@SeNPs-APS could be transformed to SeCys_2_ and Se (IV) to execute the bioactivity.

As the form SeCys_2_ is usually present in the active site of enzymes, it plays a critical role in regulating biological processes. Any protein that contains SeCys_2_ incorporated into the polypeptide chain is defined as selenoprotein [51]. There are at least twenty-five human selenoproteins and some of them have been identified as antioxidant enzymes to suppress oxidative stress-induced damage [52], including glutathione peroxidase (GSH-Px), thioredoxin reductases (TrxR), selenoprotein K (SelK) and selenoprotein T (SelT). Inspired by this, we firstly investigated the antioxidant ability of TSIIA@SeNPs-APS in vivo by measuring the activity of GSH-Px in the spinal cord tissue. As shown in Fig. 6E, the activities of GSH-Px in the SCI group was obviously lower than that in the sham group while it was enhanced remarkably after the treatment with SeNPs and TSIIA@SeNPs-APS, respectively. Malondialdehyde (MDA), formed by lipid peroxidation, is considered as a sign of oxidative stress [53]. Hence, the content of MDA was also assessed in the spinal cord tissue. As shown in Fig. 6F, the MDA content were significantly lower in the SeNPs and TSIIA@SeNPs-APS group than that in the SCI group. These results demonstrated significant antioxidant ability of TSIIA@SeNPs-APS with a better effect than that of SeNPs, which were consistent with those observed in vitro. Therefore, we further determined the effects of TSIIA@SeNPs-APS on the expression of antioxidant selenoproteins by western blot and quantitative real-time PCR (qPCR). The western blot results indicated that the expression of GPX4, TrxR1, TrxR2 and SelT were significantly declined in SCI group, and reversed by the TSIIA@SeNPs-APS. The qPCR results in Fig. 6H also indicated that TSIIA@SeNPs-APS treatment effectively enhanced the mRNA expression of GPX4, TrxR1, TrxR2 and SelT compared with the SCI group, which were consistent with the western blot results and suggested that TSIIA@SeNPs-APS played an antioxidant role by up regulating GPX4, TrxR1, TrxR2 and SelT. We also observed that the protein expression of GPX2 and SelK were increased in SCI group, and restored in the TSIIA@SeNPs-APS group, which may be due to stress response. The genes of GPX2 and SelK were down-regulated in the SCI group while reversed in the TSIIA@SeNPs-APS group. Based on these results, we concluded that TSIIA@SeNPs-APS exerted a neuroprotective effect for SCI rats via metabolized to SeCys_2_ and regulating antioxidant selenoproteins to resist oxidative stress-induced damage in spinal cord.

In addition, the histological analysis of various organs was observed to study the toxicities by H&E staining. No apparent abnormal pathological changes were found in all treatment groups, which demonstrated the biosafety of TSIIA@SeNPs-APS (Fig. 6I).

## Conclusions

In this study, TCM active ingredients-based SeNPs surface decorated with APS and loaded with TSIIA (TSIIA@SeNPs-APS) were successfully synthesized under the guidance of the compatibility theory of TCM. Our findings can be summarized as follows. Firstly, such design improved the bioavailability of APS and TSIIA with the benefits of high stability, efficient delivery and highly therapeutic efficacy for SCI treatment illustrated by an improvement of antioxidant protective effects of APS and TSIIA. Secondly, the mechanisms of the protective effects of TSIIA@SeNPs-APS against oxidative stress-induced cytotoxicity in PC12 cells were through inhibiting excessive ROS production, so as to alleviate mitochondrial dysfunction to reduce cell apoptosis and S phase cell cycle arrest, and finally promote cell survival. Finally, TSIIA@SeNPs-APS can protect spinal cord neurons in SCI rats by enhancing GSH-Px activity and decreasing MDA content, which was possibly via the metabolism of TSIIA@SeNPs-APS to SeCys_2_ and regulating antioxidant selenoproteins to resist oxidative stress-induced damage. These findings suggested that TSIIA@SeNPs-APS exhibited promising therapeutic effects in the anti-oxidation therapy of SCI, which paved the way for developing the synergistic effect of TCM active ingredients by nanotechnology to improve the efficacy as well as establishing novel treatments for oxidative stress-related diseases associated with Se metabolism and selenoproteins regulation.

## Methods

### Synthesis of TSIIA@SeNPs-APS

1 mL APS solution (20 mg/mL) and 0.25 mL TSIIA solution (1 mg/mL) was mixed with 2 mL Na_2_SeO_3_ solution (20 mM) at room temperature. Then, 2 mL Vc solution (40 mM) was added slowly into the mixture and diluted with Milli-Q water into a total volume of 10 mL. The mixture was stirred for 12 h and dialyzed against Milli-Q water for 24 h. The concentration of Se in TSIIA@SeNPs-APS was measured by atomic fluorescence spectrometer and the concentration of APS and TSIIA in TSIIA@SeNPs-APS were detected by HPLC.

Couramin-6-labelled TSIIA@SeNPs-APS for evaluation of cellular uptake and intracellular trafficking was synthesized according to the protocol of our previous study [54]. Briefly, coumarin-6 was added into the mixture of Na_2_SeO_3_, APS and TSIIA in the dark before the addition of Vc.

### Characterization of TSIIA@SeNPs-APS

The microstructure of TSIIA@SeNPs-APS was characterized by the TEM and AFM. The particle sizes and Zeta potential were analyzed by a Malvern Zetasizer Nano ZS. The chemical structure was characterized by XPS, FT-IR and UV–Vis.

### Cellular uptake of TSIIA@SeNPs-APS

PC12 cells were treated with couramin-6-labelled TSIIA@SeNPs-APS for multiple times (0.5, 1, 2, 4 and 6 h). The fluorescence intensity of couramin-6-labelled TSIIA@SeNPs-APS in PC12 cells was measured by flow cytometry as described in previous study [55].

To investigate the mechanisms of endocytosis of TSIIA@SeNPs-APS, PC12 cells were pretreated with the endocytic inhibitor CPZ (10 μg/mL) or DOG (50 mM) or nystatin (10 μg/mL) or dynasore (80 μM) for 1 h before incubate with couramin-6-labelled TSIIA@SeNPs-APS for 2 h. Then, cells were lysed by 0.2 M NaOH (containing 0.5% Triton X-100) for 10 min. The fluorescence intensity was determined at 458/520 nm by BioTek Cytation5.

### Intracellular trafficking of TSIIA@SeNPs-APS

PC12 cells were stained with DAPI and Lysotracker (80 nM) for 2 h. Then, cells were treated with 2.5 μM couramin-6-labelled TSIIA@SeNPs-APS and the tracks were visualized at different time points (0.5, 1, 2, 4 and 6 h) using a fluorescence microscopy.

### Cell culture and viability

PC12 cells were purchased from ATCC (Manassas, VA) and cultured in DMEM medium with 10% FBS, 1% penicillin–streptomycin at 37 °C in an atmosphere with 5% CO_2_. The cells were seeded in a 96-well plate (10 ×104 cells/mL) and the cell viability was measured by CCK-8 assay after the following treatment.

For evaluating the safe dosage range of TSIIA@SeNPs-APS on PC12 cells, the cells were treated with different concentrations of TSIIA@SeNPs-APS for 24 h.

For evaluating the effects of t-BOOH on cell viability reversed by TSIIA@SeNPs-APS, the cells were pretreated with 100 μM t-BOOH for 2 h and then treated with SeNPs, SeNPs-APS, TSIIA@SeNPs-APS, APS and TSIIA for 24 h, respectively.

### Cell cycle, apoptosis and ΔΨm analysis by flow cytometry

PC12 cells were seeded in a 6-well plate (10 × 10^4^ cells/mL). After 24 h, the cells were pretreated with 100 μM t-BOOH for 2 h and then treated with SeNPs, SeNPs-APS, TSIIA@SeNPs-APS for 24 h, respectively. After the following staining, the cells were detected by flow cytometry.

For cell cycle analysis, PC12 cells were stained with PI at room temperature for 30 min after fixed in 70% ethanol at −20 °C for 12 h.

For apoptosis analysis, PC12 cells were stained with Annexin V and PI according to the protocol of Annexin V-FITC/PI apoptosis detection kit.

For ΔΨm analysis, PC12 cells were stained with JC-1 (10 μg/mL) at 37 °C for 30 min. JC-1 fluorescence images of PC12 cells were obtained by fluorescence microscope.

### ROS scavenging efficiency

For ABTS^•+^ scavenging efficiency evaluation, ABTS solution (5 mM) was reacted with manganese dioxide to obtain ABTS^•+^ solution. Then, the ABTS^•+^ solution was diluted with PBS into ABTS^•+^ working solution with absorbance of 0.60 ± 0.02 at 734 nm wavelength and incubated with SeNPs, SeNPs-APS and TSIIA@SeNPs-APS (300 μM) for 2 h, respectively. The absorbance was measured by a microplate spectrophotometer at 734 nm.

For H_2_O_2_ scavenging efficiency evaluation, H_2_O_2_ solution (100 μM) was incubated with SeNPs, SeNPs-APS and TSIIA@SeNPs-APS (300 μM) for 24 h, respectively. The H_2_O_2_ content was detected using a Hydrogen Peroxide Assay Kit (Beyotime Biotechnology Co., Ltd., China).

For •OH scavenging efficiency evaluation, FeSO_4_ solution (9 mM) was incubated with H_2_O_2_ solution (8.8 mM) for 10 min to obtain •OH solution. Then, the •OH solution was incubated with SeNPs, SeNPs-APS and TSIIA@SeNPs-APS (300 μM) for 30 min, respectively. After that, the mixture was incubated with salicylic acid solution (9 mM) for 20 min and the absorbance was measured by a microplate spectrophotometer at 510 nm.

### Intracellular total ROS detection

PC12 cells were seeded in a 96-well plate (30 × 10^4^ cells/mL). After 24 h, the cells were pretreated with SeNPs, SeNPs-APS, TSIIA@SeNPs-APS (2.5 μM) for 24 h, respectively. Next, PC12 cells were stained with DCFH-DA (10 μM) at 37 °C for 30 min. Then, the cells were treated with t-BOOH (100 μM) before the fluorescence intensity was determined at 458/520 nm by BioTek Cytation5. DCFH-DA fluorescence images of PC12 cells were obtained by fluorescence microscope.

### Animal model of SCI

2 months old female Sprague–Dawley rats weighing 200 to 220 g were purchased from Guangdong Medical Laboratory Animal Center (Guangdong, China). The SCI rat model was performed as described in previous study [56]. Briefly, the rat was anaesthetized and a laminectomy was carried out at T10 under aseptic conditions. The spinal cord injury was caused by the parameters of 0.8 mm impact depth, 1.0 m/s impact speed and 85 ms retention duration from the PinPoint^™^ precision impactor device (PCI3000-2, Hatteras Instrument, USA). Rats in sham group were only subjected to surgery of laminectomy but no impact injury to the spinal cord. Finally, the wound was sutured in layers. As prophylactic for infections, penicillin was given by intramuscular injection, with dose of 16 × 10^4^ U per animal, once a day, 3 consecutive days from the day of surgery. Bladder care was conducted twice daily until bladder function resumed.

The rats were randomly assigned to six groups: sham, SCI, APS, TSIIA, SeNPs and TSIIA@SeNPs-APS group. SeNPs and TSIIA@SeNPs-APS were administered intraperitoneally at a dose of 1 mg/kg (calculated by Se concentration). APS and TSIIA were administered intraperitoneally with the same concentration of that in TSIIA@SeNPs-APS. Rats in the sham and SCI group were received the same amount of saline. All rats underwent treatment once a day, for 21 consecutive days.

On the 21th day after treatment, behavioral assessments were performed before all rats were sacrificed. Then, the spinal cord, heart, liver, spleen, lungs and kidneys were harvested for follow-up experiments.

### Behavior evaluation

Behavior evaluation was conducted using the BBB test, inclined plane test, footprint analysis and hindlimb movement analysis. The BBB score was calculated as mentioned previously [31]. The score of 0 to 21 was assigned to the rats with completely lost hindlimb movement to normal movement. The inclined plane test was conducted by acquiring the most inclined angle when the rat was able to maintain standing for five seconds. Footprint analysis was conducted by applying black dye to the rat’s hindlimbs before the rats traversed a narrow runway (1 m–long) lined with white paper to record the hindlimbs tracks. Hindlimb movement analysis was conducted by video recordings when the rat was walking through a runway. The motion of stamp-propel-lift-swing-stamp were recorded.

### Tissues staining

Fixed spinal cord tissue was subjected to dehydration, dipping in wax, embedding and sectioning at a thickness of 4 μm and then H&E, Nissl and TUNEL/DAPI double-staining were performed as mentioned previously [57]. The observation of the stained sections was conducted using the light microscope. In addition, the main organs including heart, liver, spleen, lungs and kidneys after fixation were also collected for H&E staining to evaluate the toxicity of TSIIA@SeNPs-APS in vivo.

### Monitoring of TSIIA@SeNPs-APS responding to the injured spinal cord microenvironment

Cold PBS supplemented with protease inhibitors were added to fresh spinal cord tissue. Then, each tissue was homogenized using a Tissuelyser-24 (Shanghai Jingxin Co., Ltd., China) with the parameters of 35 Hz and 60 s for three times to obtain the spinal cord homogenates. TSIIA@SeNPs-APS were incubated in injured spinal cord homogenates at 37 °C for 24 h. Images were taken by TEM.

### Metabolism analysis of TSIIA@SeNPs-APS

Metabolism analysis of TSIIA@SeNPs-APS were measured as previously reported [50] . Briefly, fresh spinal cord tissue was digested with proteinase K and trypsin. Then, the sample was centrifuged and the supernatant was collected for detecting the metabolism of TSIIA@SeNPs-APS using HPLC-ICP-MS. The standards of Se-related metabolic products including SeCys2, MeSeCys, Se (IV), SeMet, Se (VI) were used for quantifying the metabolic products of TSIIA@SeNPs-APS in spinal cord. 10 mM citric acid solution (pH 4.58) was used as mobile phase with the flow rate at 0.6 mL/min.

### *Antioxidant ability analysis of TSIIA@SeNPs-APS *in vivo

The antioxidant ability of TSIIA@SeNPs-APS in vivo by measuring the activity of GSH-Px and the content of MDA in the spinal cord. Briefly, the spinal cord homogenates were obtained by RIPA Lysis Buffer containing protease inhibitors and the protein concentration was examined by a BCA protein assay kit. Then, spinal cord homogenates were examined to detect the activity of GSH-Px and the content of MDA using the corresponding assay kit (Beyotime, China) according to the specification method.

### Western blot analysis

Expression of different proteins in vitro and in vivo were analyzed by western blot. Proteins were extracted by RIPA Lysis Buffer with protease inhibitors and then separated on SDS-Page and transferred to PVDF membranes (Millipore, USA), which were then blocked and incubated with the specific primary antibody. Next, the membranes were incubated with secondary antibodies. Protein bands were visualized by ECL kit (Millipore, USA) before imaged by a Tanon 5200 imaging system. All bands were quantified by ImageJ.

### Expressions of selenoproteins by qPCR analysis

Total RNA was isolated from the spinal cord tissues by TRIzol reagent (Invitrogen, USA) and transcribed to cDNA by an Evo M-ML V RT Premix Kit (Accurate Biotechnology, China). The qPCR was performed by using SYBR Green qPCR Master Mix (Bimake, USA) on a CFX ConnectTM Real-Time PCR Detection System (Bio-rad, USA). β-actin was used for normalization. Relative mRNA expression was analyzed using the 2^−ΔΔCt^ method. (Additional file 1: Table S1).

## Supplementary Information

**Additional file 1: Table S1** Sequences of the qPCR primers used in this study. **Figure S1** ROS scavenging efficiency of (1) APS, (2) TSIIA and (3) TSIIA@SeNPs-APS by ABTS method

## Data Availability

The datasets used and/or analysed during the current study are available from the corresponding author on reasonable request.
