# Supplementary material for: Traditional Chinese medicine active ingredients-based selenium nanoparticles regulate antioxidant selenoproteins for spinal cord injury treatment
Source: J Nanobiotechnology. 2022 Jun 14;20:278. doi: 10.1186/s12951-022-01490-x (PMC9195429; doi:10.1186/s12951-022-01490-x)
Supplement: Supplementary file 1 — Additional file 1: Table S1 Sequences of the qPCR primers used in this study. Figure S1 ROS scavenging efficiency of (1) APS, (2) TSIIA and (3) TSIIA@SeNPs-APS by ABTS method [file 12951_2022_1490_MOESM1_ESM.docx]

**Supplementary Information**

**For**

**Traditional Chinese Medicine Active Ingredients-based Selenium Nanoparticles Regulate Antioxidant Selenoproteins for Spinal Cord Injury Treatment**

Siyuan Rao^1,2#^, Yongpeng Lin^1,2#^, Rui Lin^2^, Jinggong Liu^2^, Hongshen Wang^2^, Weixiong Hu^2^, Bolai Chen^1,2*^, Tianfeng Chen^3*^

**Table S1. Sequences of the qPCR primers used in this study**

**Genes Forward primer (5ʹ→3’) Reverse primer (5ʹ→3’)**

GPX2 TGTCAATGGGCAGAATCAGCATCC AGGGAGAATGGGTCGTCATAAGGG

GPX4 ATAAGAACGGCTGCGTGGTGAAG TAGAGATAGCACGGCAGGTCCTTC

TRXR1 CACGGATGAGGAGCAGACCAATG CATACAGCCTCTGAGCCAGCAATC

TRXR2 GGGAGGGCAGCAGAACTTTGATC TAGTCAGCCACAGCCACCTTCC

SelK CAGGTGTTAGACAGCCGGAATCAG CCCCGTAGCCTCTTCTTTTCTTCAC

SelT CCGCTGCTCAAGTTTCAGATTTGTG GTCTGGATACCGCTGGCTAATAACC


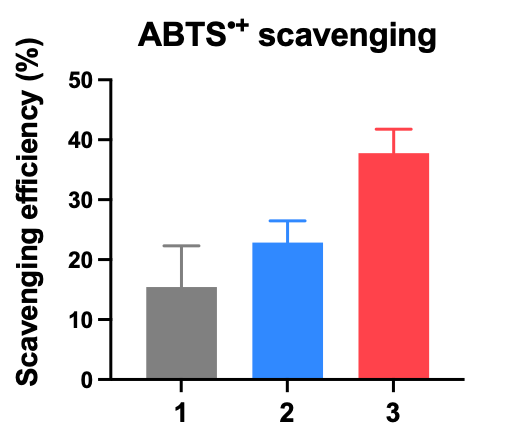


**Figure S1.** ROS scavenging efficiency of (1) APS, (2) TSIIA and (3) TSIIA@SeNPs-APS by ABTS method.
